# Current situation of midwives in indonesia: Evidence from 3 districts in West Java Province

**DOI:** 10.1186/1756-0500-3-287

**Published:** 2010-11-08

**Authors:** Peter Heywood, Nida P Harahap, Mimin Ratminah, Elmiati

**Affiliations:** 1 Menzies Center for Health Policy, University of Sydney, Australia; 2 Jalan Bukit Dago Selatan, Bandung, West Java Province, Indonesia; 3 Jalan Pratista Raya, Amtapami, Bandung, West Java Province, Indonesia; 4 Elmiati, Politeknik Kesehatan, Departemen Kesehatan, Bandung, West Java Province, Indonesia

## Abstract

**Background:**

The village midwife is a central element of Indonesia's strategy to improve maternal and child health and family planning services. Recently there has been concern that the midwives were not present in the villages to which they had been assigned. To determine the extent to which this was the case we conducted a field-based census and survey of village midwives in three districts in West Java Province, Indonesia.

**Findings:**

In June 2009 we interviewed a random sample of village midwives from three districts - Ciamis, Garut and Sukabumi - in West Java Province. Trained interviewers visited all villages represented in the sample to interview the midwives. We also obtained information about the midwives and their professional activities in the last year.

Thirty percent of village midwives had moved to another location in the 12 months between the end of 2008, when the sampling frame was constructed, and December 2009 when the survey was conducted; most had moved to a government health center or another village. Of those who were present, there was considerable variation between districts in age distribution and qualifications. The total number of services provided was modest, also with considerable variation between districts. The median number of deliveries assisted in the last year was 64; the amount and mix of family planning services provided varied between districts and were dominated by temporary methods.

**Conclusions:**

Compared to an earlier survey in an adjacent province, the village midwives in these three districts were younger, had spent less time in the village and a higher proportion were permanent civil servants. A high proportion had moved in the previous year with most moving to a health center or another village. The decision to move, as well as the mix of services offered, seems to be largely driven by opportunities to increase their private practice income. These opportunities are greater in urban areas. As urbanization procedes the forces drawing village midwives away from the village are certain to strengthen. This will require a reassessment of the original service model embodied in the village midwife concept and a new approach to reducing maternal mortality.

## Background

The village midwife (*bidan di desa*) is a central element of Indonesia's strategy to improve maternal and child health and family planning services. The original training for midwives was through the *Sekolah Bidan *(SB), a one-year program which students entered at the end of junior high school. The SB Program was closed in 1984 and no midwives were trained for 5 years until, in 1989, as part of the response to the international safe motherhood conference in Nairobi [1], the government started the *Program Pendidikan Bidan (PPB) - *entrants to this program, which was closed in 1998, had a basic nursing qualification. The PPB was replaced in 1998 by the current *Akademi Bidan (Akbid) *Program, a three-year course which students enter at the end of their senior high school year. The current workforce contains midwives trained in all three programs with the proportion trained in the *Akbid *Program increasing over time.

Midwives in the public sector are employed as permanent civil servants (PNS), and through contracts with the central, provincial and district governments and with health facilities; they work in hospitals, health centers, auxiliary health centers and village midwife posts. Midwives are also found in the private sector in hospitals, maternity hospitals, maternity clinics, treatment clinics (balai pengobatan) and in private practice on their own account. Public sector midwives have private practice rights and most also engage in private practice after formal work hours. A study of human resources for health in 15 districts in Java (5 in East Java, 5 in Central Java and 5 in West Java) found that in 2006 56% of midwives were permanent civil servants, 34% were employed under various forms of contract (including PTT - a scheme originally introduced in 1992 to employ doctors on limited term contracts in the public sector. In 1994 the scheme was extended to include midwives. The PTT scheme continues for midwives even though it was discontinued for doctors in 2007), and 10% were in private practice. In the 10 districts in West and Central Java, 95% worked for the government - 54% were village midwives and 41% worked in a health center - and 5% were in the private sector [2].

The village midwife program (*bidan di desa*) commenced in 1989 with the aim of providing maternal and child health and family planning services, primarily to rural areas. The initial goal was to place a midwife in every village in the country but there are many areas in which this has not been achieved [3,4].

In the course of our earlier work [2] on human resources at the district level some health administrators expressed two concerns about the village midwife program which was receiving added emphasis through the Millennium Development Goals: first, that they were unable to place a midwife in every village - indeed, reports indicate that approximately 40% of villages currently have a midwife and that there is considerable variation between provinces and districts [5]; and second, concerns in some districts that many midwives were no longer in the villages but have moved to areas where more lucrative private practices could be established while retaining a nominal village location. These concerns come at a time when the central government is engaged in a major expansion of the midwife scheme in two ways: PTT midwives employed before 2005 had already been offered the chance to become permanent public servants; and the various levels of government are each considering new measures to increase the number of village midwives [Heywood P, Harahap NP, Aryani S. Recent changes in human resources for health and health facilities at the district level in Indonesia: evidence from 3 districts in Java. Unpublished].

The objective of the study was to determine the extent to which village midwives are actually working in the village in which they are recorded as being located in three districts of West Java Province, Indonesia. At the same time we collected basic information about the village midwives and the services they provide. The results are reported here.

## Methods

To achieve the study objective we made a physical, field-based census and survey of a sample of village midwives, PNS or PTT, in three of the five districts of West Java Province, Indonesia, in which we had earlier enumerated all health care providers (doctors, nurses and midwives) on the basis of government personnel records. For each midwife contacted we recorded information about their age, marital status, qualifications, length of time in the village, employment status, in-service training in the last year, and professional services provided (birth deliveries, contraceptive services) in the previous year. This information was recorded in a pre-coded questionnaire.

The sample for this study was selected as follows. In July 2007, as part of a study of the functioning of the health system at the district level, we enumerated all village midwives present in 5 districts (Ciamis, Cirebon, Garut, Sukabumi, Subang) in West Java Province, Indonesia at the end of 2006 [2]. In order to describe changes in health personnel and facilities in the intervening two years, this list was updated at the end of 2008 - the results are reported in [Heywood P, Harahap NP, Aryani S. Recent changes in human resources for health and health facilities at the district level in Indonesia: evidence from 3 districts in West Java Province. Unpublished.] In December 2009 we interviewed a random sample of village midwives from three of the districts - Ciamis, Garut, and Sukabumi. The random sample of 55 village midwives was drawn in each of the 3 districts from the updated list (as at the end of 2008) using systematic random sampling. Under the supervision of one of the authors (MR), a senior midwife familiar with the province and the three districts, trained interviewers then visited all villages represented in the sample to interview the midwives selected.

If a midwife in the sample was no longer living in the village the interviewer ascertained from the village leaders the reasons the midwife had moved. This missing midwife was then replaced by the next midwife on the list of midwives used as the sampling frame. The replacement midwife was visited and information collected using the questionnaire discussed above.

All data were entered to a computer twice. The two files were compared and differences resolved by referring back to the interview form and telephone follow-up with the midwife if necessary. To preserve confidentiality the name of each midwife and of the village in which she worked was de-linked from the remainder of the survey results. Hard copy of survey forms is retained by one of the authors (NH) in a locked filing cabinet. The data were analyzed using STATA version 9.

The study was carried out with the approval of the Provincial Health Office of West Java Province.

## Results

In all three districts, at least a quarter of those sampled had moved to another location in the 12 months between the end of 2008 (when the list of village midwives in each district was updated) and the survey at the end of 2009; almost 40% had done so in Garut.

Overall, 30% had moved (Table 1). The reasons for moving are shown in Table 2. Of the 50 midwives who had moved, 40% had moved to a (government) health center, 18% to another village, 10% had been promoted and 10% had gone for further study; of the 50 who had moved, almost 80% were still within the health system but no longer in the village in which they had been one year earlier.

**Table 1:** Midwives in sample who had moved from the village, by district.

District	Number sampled	Number moved	% moved
Ciamis	53	15	28
Garut	55	21	38
Sukabumi	56	14	25
			
3 districts	164	50	30

**Table 2:** Reasons for move from village.

Reasons not at village	Frequency	Percent
Moved to health center	20	40
Moved to another village	9	18
Promoted	5	10
Further study	5	10
Moved to Family Planning Agency	1	2
Pregnant	1	2
Retired	1	2
Died	1	2
Other	7	14
		
Total	50	100

Each of the 50 midwives in the original sample who were no longer in the village in which government records indicated they lived a year earlier was replaced by the next midwife on the list that formed the sampling frame. Thus, the total sample across the three districts was 164 and the results for them are shown in subsequent tables.

There was statistically significant variation in the characteristics of the sample between districts for age, marital status, qualifications, years living in the village, and employment status (Table 3). Overall, 26% of the midwives were < 30 years of age with marked differences in age distribution between the districts - in Ciamis only 8% were below 30 years of age whereas in Garut and Sukabumi it was 31% and 38%, respectively. Although most were married, the lower proportion married in Sukabumi reflects the younger age distribution. The younger age distribution is also reflected in two other characteristics: first, the higher proportion of midwives with D3 qualification (31% overall) in Sukabumi (43%) compared to 36% and 13% in Garut and Ciamis, respectively; and secondly, the length of time in the village - 52% less than 5 years in Sukabumi versus only 17% in Ciamis and 45% in Garut. Across the three districts 73% were permanent civil servants with the proportion rising to more than 90% in Ciamis, whereas the other two districts made more use of contract workers, in Garut mostly longer term contracts and in Sukabumi mostly shorter term contracts at the local level.

**Table 3:** Age, marital status, initial qualifications, years in village and employment status in Ciamis, Garut, Sukabumi districts, Indonesia, 2009.

	Ciamis	Garut	Sukabumi	Total	Chi sq p
	%	%	%	%	
Age					0.001
20-29	8	31	38	26	
30-39	81	58	46	62	
40-49	11	11	16	12	
N	53	55	56	164	
					
Marital status					0.005
Married	98	95	84	92	
Not married	2	5	16	8	
N	53	55	56	164	
					
Initial qualifications					0.002
Sekolah bidan	4	0	2	2	
D1	83	64	55	67	
D3	13	36	43	31	
N	53	55	56	164	
					
Years in village					0.001
< 1.0	0	10	24	12	
1.0-1.9	4	14	15	11	
2.0-4.9	13	21	13	15	
5.0-9.9	18	14	15	15	
10.0-19.9	65	41	33	46	
N	46	49	54	149	
					
Employment status					0.001
Permanent	92	62	64	73	
Contract	8	38	36	27	
N	53	55	56	164	

Chi sq - difference in distributions between districts assessed using Fisher's exact test.

In the previous 12 months 40% of the village midwives had received in -service training on family planning, 18% on normal delivery care, 25% on resuscitation of neonates and only 4% on life saving skills.

For services, there is considerable and statistically significant variation between districts for all services delivered (Table 4). The median number of deliveries per year was 64 overall with 56 in Ciamis and 96 in Sukabumi. Sukabumi also had the highest median number of contraceptive pill services, twice the number of Ciamis. On the other hand, Garut had the highest number of contraceptive injections, more than 3 times the number given in Sukabumi and almost 5 times the number in Ciamis. The uptake of IUDs and implants was low in all three districts.

**Table 4:** Services provided by village midwives in Ciamis, Garut, Sukabumi districts, Indonesia, 2009

Median services in last 12 months	Ciamis	Garut	Sukabumi	Total	K Wallis p
Deliveries (p25, p75)	56	57	96	64 (48, 100)	< .001
N	52	55	56	163	
Contraceptive pill (p25, p75)	38.5	65	77	60 (28,120)	< .001
N	52	53	56	161	
Contraceptive injections (p25, p75)	105	500	150	233 (97, 527)	< .001
N	41	50	37	128	
Intrauterine device (p25, p75)	5	10	6	6 (3, 15.5)	< .05
N	45	45	38	128	
Implants (p25, p75)	2	10	8	6 (2, 13.5)	< .001
N	35	32	29	96	

## Discussion

Taken across the 3 districts, these results indicate a midwife workforce that is relatively young (26% < 30 years of age), many of whom have thus been in the village for a short period of time (38% in village < 5 years overall, but only 17% in Ciamis) and who are increasingly likely to have graduated from a three-year training program (31%).

Nevertheless, there is considerable variation between districts with those in Ciamis being older, and with more time in the village compared to Sukabumi where the midwives are much younger.

In terms of overall characteristics, the main point of comparison for these results is with those of Makowiecka et al [6] who studied 361 village midwives in two districts of Banten Province (adjacent to West Java province) in 2005. Only 4% of their sample had been in a village for less than 5 years compared to more than half the midwives surveyed here. Only 54% of the Banten sample were permanent civil servants compared to 73% in the current West Java sample; and only 3% of the Banten sample had 3 years pre-service training (D3) compared to 31% in the West Java sample.

Midwives were originally placed in the villages as part of Indonesia's response to calls for Safe Motherhood Programs after the Nairobi conference in 1987. The midwives had two main tasks - to provide skilled delivery assistance either in the mother's home or in their own home; and to be the frontline agents of the national family planning program. The midwives were granted private practice rights with the long term aim of sustaining themselves as they provided assistance to the village population.

The demand for the services of these village midwives is influenced by the alternatives available. For birth deliveries, the alternatives are to deliver with relatives, with a traditional midwife, or with a village midwife, and, in some cases, both. With respect to delivery assistance, the median number of deliveries per year was 64, compared to 40 deliveries per year for village midwives in the Banten study. This raises the question of the minimum number of deliveries necessary for maintenance of skills at an adequate level. Scotland and Bullough [7] consider that 100 to 125 normal deliveries is an optimal annual workload for obstetricians. Makowiecka et al [6] point out that while there is no internationally agreed minimum number of deliveries for a midwife to maintain her skills, application of the same recommendation to Indonesian midwives means that many have delivery volumes that fall "well below optimal levels, and their capacity to manage complications and recognize the need for referral may be compromised because they come across these situations so infrequently.". Others have noted the problems with quality of services [8] and the need for improved skills [9] of midwives in Indonesia.

For family planning services there are many more options which include integrated health posts (posyandu), health centers and subsidiary health centers, the private practices of doctors, nurses, and other health staff, nursing homes and hospitals. The mix of services within a district varies according to total area, population density, infrastructure and the number of health staff. The family planning services provided by these midwives place heavy reliance on temporary, rather than permanent, contraceptive methods. This reflects a mix of supply side issues which mean that, in order to increase income, midwives are more likely to offer temporary methods which involve repeat services (the pill, 1 month injections or, less likely, 3 month injections) in preference to IUDs, implants and sterilization [10]. At the same time women may also be expressing their own preferences for methods that are not so long term or permanent.

Consistent with the views of district health administrators, there is considerable mobility in this workforce and those who are located in the village are often not there for appreciable periods of time. Thirty percent of the village midwives had moved within one year of verifying the sample frame with considerable differences between districts - almost 40% of the sample in Garut and 25% in Sukabumi. The most common reason for moving was to take up a position in a health center or promotion or further study - these 3 reasons alone accounted for 60% of all moves. A further 18% moved to another village. Thus, of the 50 who moved more than half had moved to positions out of the village altogether and were unlikely to return there. Less than 20% of those who moved were still in a village, even if it was a different one. Further, those who are based in a village may spend considerable time away from it. Thus, in Sukabumi, of those actually interviewed, more than one-quarter (15/57) were not present at the time of the first visit by the interviewer. Of those away at the time of the first visit, 40% were at part-time training, 40% were away on private business; only 20% were away delivering health care.

The move to a puskesmas is largely driven by preference for shorter official working hours, improved status, and the opportunity to establish a more lucrative private practice in, or closer to, urban areas. As urbanization procedes (more than half the Indonesian population now lives in urban areas [11]) the tendency to move to urban areas is likely to increase.

Notwithstanding the variation between districts, the picture of village midwives that emerges here is of a solo private practitioner (as has been the case since the beginning), an increasing proportion of whom are young with more years of training but skills of uneven quality, the majority with substantial public subsidy from their permanent civil service salaries, many of whom may spend appreciable periods away from the village, do not provide large quantities of services and have a modest income from this source. The incentive structure means that the mix of services they do provide is often designed to optimize their own income rather than meet the longer term needs of their clients and that the goal for many is to move to urban areas, and failing that to a larger village, where private practice is more lucrative. The preferred way to achieve this is through transfer to a government health center. As urbanization procedes the forces drawing village midwives away from the village are certain to strengthen requiring a reassessment of the original service model embodied in the village midwife concept and a new approach to reducing maternal mortality.

## Competing interests

The authors declare that they have no competing interests.

## Authors' contributions

PH designed the study, analyzed data, and drafted the paper. NPH contributed to study design, interpretation and assisted with drafting the paper. MR supervised data collection and contributed to data interpretation. Elmiati carried out data entry and assisted with interpretation of results. All authors read and approved the final version of the paper.
